# Sex differences in brain plasticity: a new hypothesis for sex ratio bias in autism

**DOI:** 10.1186/s13229-015-0024-1

**Published:** 2015-06-05

**Authors:** Laurent Mottron, Pauline Duret, Sophia Mueller, Robert D Moore, Baudouin Forgeot d’Arc, Sebastien Jacquemont, Lan Xiong

**Affiliations:** Centre d’excellence en Troubles envahissants du dévelopement de l’Université de Montréal (CETEDUM), Montréal, Canada; Hôpital Rivière-des-Prairies, Département de Psychiatrie, Montréal, Canada; Centre de Recherche de l’Institut Universitaire en Santé Mentale de Montréal, Québec, Canada; Department of Psychiatry, University of Montreal, Québec, Canada; Département de Biologie, École Normale Supérieure de Lyon, Lyon, CEDEX 07 France; Institute of Clinical Radiology, University Hospitals, Munich, Germany; Athinoula A. Martinos Center for Biomedical Imaging, Department of Radiology, Massachusetts General Hospital, Charlestown, MA 02129 USA; Harvard University, Center for Brain Science, Cambridge, MA 02138 USA; Department of Health Sciences, University of Montreal, Montreal, Canada; College of Applied Health Sciences, University of Illinois, Urbana-Champaign, USA; Centre de recherche, Centre Hospitalier Universitaire Sainte Justine, Montréal, Canada; Service of Medical Genetics, University Hospital of Lausanne, University of Lausanne, Lausanne, 1011 Switzerland

**Keywords:** Autism spectrum, Sex ratio, Male bias, Synaptic plasticity, Regional plasticity, Perceptual associative cortex, Sexual dimorphism, Enhanced perceptual functioning

## Abstract

Several observations support the hypothesis that differences in synaptic and regional cerebral plasticity between the sexes account for the high ratio of males to females in autism. First, males are more susceptible than females to perturbations in genes involved in synaptic plasticity. Second, sex-related differences in non-autistic brain structure and function are observed in highly variable regions, namely, the heteromodal associative cortices, and overlap with structural particularities and enhanced activity of perceptual associative regions in autistic individuals. Finally, functional cortical reallocations following brain lesions in non-autistic adults (for example, traumatic brain injury, multiple sclerosis) are sex-dependent. Interactions between genetic sex and hormones may therefore result in higher synaptic and consecutively regional plasticity in perceptual brain areas in males than in females. The onset of autism may largely involve mutations altering synaptic plasticity that create a plastic reaction affecting the most variable and sexually dimorphic brain regions. The sex ratio bias in autism may arise because males have a lower threshold than females for the development of this plastic reaction following a genetic or environmental event.

## Review

### Introduction

Boys have a 4 to 7 higher-fold risk of developing autism than girls [1], (for a review see [2]). The ‘protective effect’ observed in females with respect to the autism spectrum (AS) remains largely unexplained [3]. X-linked variants have been explored as obvious candidates; however, the frequency of monogenic X-linked intellectual disability (ID) in AS is too low (5% to 8% of males with autism) to account for the imbalance in the AS sex ratio [4-6]. The most preeminent hypothesis for the AS sex ratio, and other sex-specific associations in autism, is the extreme male brain (EMB) theory [7]. In this paper, we propose as an alternative the enhanced plasticity hypothesis (see Table 1 for a summary of these two theories), which is consistent with the EMB theory in some aspects and inconsistent in others. We focus on sex differences in plasticity at the synaptic and regional level and hypothesize that these differences influence the threshold for the development of plastic reactions in specific brain areas involved in perception and language.

**Table 1:** **Accounts of the enhanced plasticity hypothesis and extreme male brain theory to explain sex–related features in the autism spectrum**

	**Sex-related autistic features**	**Enhanced plasticity hypothesis**	**Extreme male brain theory**
Sex ratio	For every female, four to ten males are identified with autism.	Males have a lower threshold than females for developing an enhanced plastic reaction to the same biological event. This reaction targets perceptual or language-related cerebral regions, resulting in autistic strength and social neglect.	Autism reflects ‘extreme expression of the (…) attributes of the male brain'; males require fewer biological changes than females to exhibit autism; strong empathy skills protect women against autistic social deficits.
IQ/sex ratio relationship	The excess of males increases with IQ.	Severe mutations leading to low IQ mask any sex-differences. In high functioning AS, mutations are less deleterious, preserving normal, sexually dimorphic plasticity.	Females with syndromic autism are easier to identify than those with high IQ autism because the social competence of women compensates for autistic social features.
Social & communication phenotype	AS males show more social and communication impairments than AS females.	Social symptoms result from the neglect of socially-guided behaviors caused by the domination of perceptual (AS individuals with SOD) or language (AS individuals without SOD)-guided behaviors, which are in turn favored by strong experience-dependent plasticity in males.	Social symptoms result from strong expression of the non-autistic male social behavioral phenotype, which is less driven toward the social domain than that of females.
RRBI phenotype	AS males show more RRBI than females.	Enhanced perceptual-functioning accounts for perception-based RRBI.	Some high-level RRBI are rule-based behaviors accounted for by extreme systematizing, a male strength.
		Perceptual regions are more likely to be targeted by the enhanced plastic reaction in males than in females.	
Non hormone-related genetic findings	Many genes involved in autism are related to synaptic plasticity, and some show sexually-dimorphic expression. - Deleterious and DN CNV/ SNV are more frequent in autistic females than males.	Synaptic plasticity is expressed through different mechanisms in males and females throughout life. The ‘genetic protective effect’ in females contributes to the small sex-ratio in low-IQ autism.	Genetic differences unrelated to hormonal effects are not accounted for by the EMB model.
		Causative mutations are more likely to affect or trigger a plastic reaction in males than in females, because of the lower threshold of plasticity in males.	
	AS females and low-IQ males, but not high-IQ AS have a high incidence of DN LGD mutations.	Deleterious mutations disrupt plasticity resulting in a similar phenotype, mostly composed of ‘negative’ signs, in low IQ males and females.	
Hormone-related genetic findings	A dozen sex-steroid pathway genes are associated with AS.	Sex steroids modulate plasticity mechanisms resulting from non-steroid related plasticity genes and thus can also be associated with autism.	Genetic alteration of the sex-steroid pathway modifies fetal androgens levels, causing hyper-masculinization of the autistic brain.
Brain structure	Autism-related differences overlap with sexually dimorphic structures.	Autism-related differences overlap with the most variable regions of the non-autistic brain. Differences in structure may reflect previous differences in plasticity mechanisms.	Altered brain structure in AS results from hyper-masculinization.
Brain connectivity	Connectivity patterns in AS resemble particularities of the brain network of non-autistic males.	Connectivity reflects the way networks were organized by plasticity mechanisms mainly during development. Loci of enhanced connectivity are determined by loci of enhanced variability in humans.	Connectivity pattern in autism results from hyper-masculinization of the brain networks.
Brain function	Autism-related differences overlap with sexually dimorphic patterns of activation.	Enhanced experience-dependent plasticity in associative perceptual (or language) regions, accounts for enhanced activity in autism. Given that these regions are among the most variable and plastic areas and underlie some of the most recent functions in evolution, they are likely to show group differences in activity like male/female or AS/non-AS dimorphisms.	Altered activations sometimes show the pattern females > males > AS or AS > males > females, reflecting hyper-masculinization of brain functions.
Cognition	Autistic strengths and non-autistic male strengths overlap.	This overlap is limited to some visuospatial tasks. Autistic strengths extend beyond male strengths, and manifest as enhanced perceptual functioning, even in domains where no clear male advantage is reported (for example, pitch).	AS individuals strongly express male cognitive strengths and weaknesses.
	Scores in psychological tests show the following patterns:		
	Systemizing/Autism Quotients: AS > males > females		
	Empathy Quotient:	SQ/EQ/AQ tests are not sensitive enough to distinguish male/female differences from autism/control differences.	
	Females > males > AS		
Behavioral phenotype	Autistic males present more social negative symptoms and positive repetitive symptoms.	Differences in male and female autistic phenotype result from sex differences in the target of the plastic reaction.	Phenotypic gender differences reflect the masculinization of autistic behavior.

In some domains, the two hypotheses are not mutually exclusive and represent complementary views. References: the current review, [7] and [217]. AS: Autism Spectrum; CNV: Copy Number Variant; DN: De Novo; EMB: Extreme Male Brain; IQ: Intelligence Quotient; LGD: Likely Gene Disruptive; RRBI: Repetitive, Restrictive Behaviors and Interests; SNV Single Nucleotide Variant, SOD: Speech Onset Delay.

#### Background: the role of altered synaptic and regional plasticity in autistic cognitive hyper functioning

Autism involves cognitive hyper-functioning and cortical reallocations [8], which have not yet been mechanistically related to sex. *Regional* plasticity, or *cortical reallocation* (the two terms are equivalent and will be used interchangeably in the text) correspond to the experience-dependent dedication of a cerebral region to a cognitive function that does not typically map to this region in the general population, for example cross-modal plasticity that occurs in sensory-impaired individuals [9]. Outstanding skills, including special isolated skills (SIS, clinically-defined domain-specific talents that contrast with an individual’s overall level of functioning) in everyday life and perceptual peaks (PP) of performance in laboratory settings are frequent features of autism. Some domain-specific skills, like absolute pitch, synesthesia, and hyperlexia, are so much more frequent in autistic than in non-autistic individuals that their combination may even be considered specific to autism [10]. In one study, the prevalence of SIS in a large group of 254 autistic individuals was 62.5% and that of PP (in a smaller group) was 58% (vs. 13% in controls). Performances in different low-level perceptual tasks co-vary, indicating that they may depend on a single domain-general factor [11]. The presence of an SIS in a particular modality is not associated with a PP in the same modality [12], which suggests that SIS and PP in autistic individuals involve a high level of brain plasticity, combining an experience-dependent component to genetically defined enhancement of perceptual encoding.

In addition to superior perceptual performance in many tasks, the functional allocation of some brain regions, in particular the visual associative cortex, is more extended and variable in autistic than in non-autistic individuals [13,14]. Together with idiosyncratic, individual-specific connectivity among functional regions [15], this high variability suggests regional reallocations of neuronal resources in autistic individuals. Using a quantitative meta-analysis of published functional imaging studies, we computed Activation Likelihood Estimation maps and found that activity in temporal, occipital, and parietal regions during a broad range of visual tasks was higher in autistic than in non-autistic individuals. By contrast, AS individuals exhibited less activity in the frontal cortex than non-autistic individuals. The spatial distribution of pattern differences between groups varied across processing domains. Autism may therefore be characterized by strong functional resource allocation in regions associated with visual processing and expertise [16].

The auditory modality provides further evidence for the plastic nature of autistic enhanced performance. When exposed to speech-like sounds, AS individuals with a speech onset delay showed high activity in the primary auditory cortex, whereas higher-order regions associated with language processing were highly active in AS individuals with normal language development. These findings suggest that cortical functional reallocations occurring in the most plastic brain regions, the multimodal association cortices[17], favor either perception or language, which may account for the main subgroups in the AS [18].

Enhanced performance and the autonomy of perception form the basis for the Enhanced Perceptual Functioning model (EPF) of autistic cognition [19]. This model proposes that the superior performance of autistic individuals in multiple basic perceptual tasks results from strong encoding mechanisms in a subset of low-level dimensions, associated with enhanced perceptual expertise and an atypically prominent role for perceptual mechanisms in cognitive function. Alternatively, in AS individuals without speech delay, incoming information is primarily processed by the hyper-functioning of typical language-related processes, resulting in language strengths, but not perceptual ones.

How may high regional plasticity result from mutations in AS-associated genes and microstructural brain plasticity mechanisms involved in autism? The Trigger-Threshold-Target (TTT) model of autism [20] attempts to link the EPF model to strong synaptic and regional plasticity. According to the TTT model, several genetic mutations and environmental insults involved in autism *upregulate* synaptic plasticity [21-23]. At the microstructural level, *synaptic* plasticity (SP) is the process of constructing and pruning synapses that occurs during development and the remodeling of these synapses during learning. SP stabilizes new experiences and is therefore involved in the ongoing experience-dependent adaptation of perception and memory systems. A *plastic reaction* to genetic or environmental events promotes SP especially in individuals with a low *threshold*, resulting in functional reallocations that in turn lead to maximal adaptation following network disturbance. The term *threshold* here refers to the level of disruption required for this plastic reaction to happen. Similar mutations may be associated with different thresholds that may lead to substantially different phenotypes [24], particularly in males and females [25]. Here, we describe compelling direct and indirect evidence from human genetics and transcriptomics, animal models, brain imaging, and studies of cerebral plasticity in development and adulthood, supporting the hypothesis that this plastic reaction is sex-dependent. We suggest that plasticity is involved in autistic sex-ratio bias because males have a lower threshold than females for the development of plastic reactions.

### Sex differences in synaptic plasticity: human genomic data and animal models of autism

#### Autism-associated genetic mutations involved in synaptic structure, function, and plasticity

Large-scale whole-exome sequencing (WES) and genome-wide copy-number variant (CNV) studies of autism have identified highly penetrant *de novo* gene-disrupting mutations in about 30% of simplex AS cases. Such mutations in approximately 400 genes contribute to autism [26-33]. Transcriptomic analyses have revealed that these genes are important for synaptic structures and functions, which may affect specific brain regions and neural circuits associated with learning, memory, and perception [34-39]. These include: (1) FMRP targets, that is, transcripts bound by the fragile X mental retardation protein (FMRP) which locally regulates the transport, stability, and/or translation rate of more than 850 brain mRNAs at the synapse, many of which are linked to synaptic function [32,33,40]; (2) genes involved in chromatin remodeling and those encoding chromatin modifiers [31,32]; (3) synaptic function and plasticity genes, in particular genes encoding postsynaptic density proteins [31,32,41]; (4) genes regulating transcription and splicing that are expressed preferentially in embryos [31,32,42,43]; and (5) embryonic development genes [44]. According to our model, these mutations represent the *Trigger* of a sexually dimorphic plastic reaction.

#### Sexual dimorphism in the expression of genes related to AS

The brain transcriptome of males and females differs throughout life, in particular during development. In a spatiotemporal transcriptomic study of the human brain, Kang *et al.* [43] identified 159 genes differentially expressed between males and females located on the Y (13 genes, 8.2%), X (nine genes, 5.7%), or autosomal (137 genes, 86.2%) chromosomes. The expression of many of these genes (76.7%) was higher in males than in females and notable topographical differences were observed. More genes showed sex-biased expression during prenatal development than during postnatal life, with the adult brain showing the fewest differences in expression. The largest differences were attributable to Y chromosome genes, especially *PCDH11Y*, *RPS4Y1*, *USP9Y*, *DDX3Y*, *NLGN4Y*, *UTY*, *EIF1AY*, and *ZFY*. These genes displayed constant expression across brain regions during development and life, and their functional homologues on the X chromosome were not upregulated in a compensatory manner in female brains. These profiles indicate that the main differences in brain development between the sexes happen during the prenatal period and are largely determined by the differential expression of genes on the sex chromosomes, although many other genes on autosomes may also contribute. Furthermore, in some genes the exons show sex-specific patterns of expression in the brain. Although the exact role of most of these sex-differentially expressed genes in brain development is not yet known, some are relevant to the pathogenic pathways of autism. For example, the *NLGN4X* gene is expressed in males and females at a similar level, but some of its exons are more strongly expressed in males in a developmentally regulated manner. Exon 7 and, to a lesser extent, exons 1, 5, and 6 of *NLGN4X* are gradually more expressed in males than in females, from the prenatal period to adulthood. A frameshift mutation (1186insT) was identified in exon 6 of *NLGN4* in one Swedish family with two affected brothers, one with typical autism and the other with Asperger syndrome [45]. Another 2-base-pair frameshift deletion (1253del(AG)) was also found in exon 5 of *NLGN4X* in 13 affected males with ASD and ID [46]. The R704C mutation of *NLGN4*, which is found in some autistic individuals may act by enhancing synaptic transmission [47]. The *NLGN4* gene belongs to the neuroligin family of cell adhesion molecules, which are located postsynaptically and bind to presynaptic neurexins [48] and netrins [49] to form trans-synaptic complexes. In mice, *NLGN4* is involved in the regulation of excitatory and inhibitory circuits and helps to balance the response to stimulation [50]; thus, this gene is important for reactive plasticity.

In another transcriptomic analysis of male-female differences in prefrontal cortical development, Weickert *et al*. [51] identified 14 Y and 11 X chromosome genes showing sexually-dimorphic expression, including many genes implicated in autism, such as *NLGN4Y* and *PCHD11Y*, which were highly expressed in infant males and may influence the early male-specific development of cortical brain cells. *PCDH11X*/Y is a human-specific gene pair located in the Xq21.3/Yp11.2 homologue region [52,53] mainly expressed in the brain [53,54]. Like other cadherin genes, *PCDH11X*/Y is localized at the synaptic junction and probably involved in the regionalization and functional differentiation of brain gray matter and in the establishment of neuronal connections or signal transduction at the synaptic membrane. *PCDH11X* transcripts are more abundant in females than in males [55]. However, a longitudinal study of the human prefrontal cortex showed that *PCDH11X* transcript levels were highest in newborn males, decreased throughout childhood, and were equally low in adults of both sexes [51]. Duplication and deletion of this region are associated with both developmental dyslexia [56] and non-syndromic language delay [57].

Altogether, these findings show that developmentally and spatially regulated differences in gene- and exon-level expression exist between male and female brains in specific regions and may have irreversible effects on cerebral architecture and plasticity. Therefore, sex differences in the temporal and spatial transcriptomic profile of the human brain during early development may modulate an individual’s vulnerability and reactivity to genetic and environmental perturbations. Spatial differences may determine the *target* regions of a plastic reaction, and temporal differences may influence the developmental course of its phenotypic manifestation.

#### High mutational burden in autistic females

A study of more than 9,000 dizygotic twins from population-based cohorts showed that siblings of autistic females exhibit significantly more autistic traits than siblings of autistic males [58-60], suggesting that female patients carry a higher ‘genetic load’ than male patients. Studies of chromosomal structural variation showed that *de novo* CNV are more common in autistic females than in autistic males and that these CNV disrupt more genes in females than in males [61-63]. Data on the following microdeletions in *SHANK1* suggest that the penetrance of AS in these CNV carriers is sex-biased: four male carriers from the same family and another unrelated male carrier of a different microdeletion presented high-functioning autism, whereas two female relatives carrying the same microdeletion showed anxiety but did not meet the diagnostic criteria for AS [64]. In a large CNV analysis of autistic individuals and their families, Pinto *et al*. [65] found that autistic females were more likely to have highly penetrant CNV and were twice as likely to have exonic deletions involving FMRP targets than autistic males.

In a cohort of 15,585 probands with neurodevelopmental disorders, Jacquemont *et al*. [66] found that deleterious autosomal CNV were more common in females than in males. Furthermore, in an independent AS cohort of 762 families, females had three times more deleterious autosomal CNV and many more unique deleterious single-nucleotide variants (SNV) than males. The effect of autosomal SNV was also substantially more severe in autistic females than in autistic males. Consistent with the notion of a protective effect in females, inherited deleterious CNV and SNV in AS individuals and those with other neurodevelopmental conditions are preferentially of maternal origin. Two recent WES analyses of AS confirm this observation [31,32]. These studies show that both autistic females and males with a low IQ have a high incidence of *de novo* (DN) likely gene disruptive (LGD) mutations. However, there were few DN LGD mutations in high-functioning males with AS. These observations suggest that gene disruptive variants, which have been the focus of recent exome studies, are strongly associated with IQ and do not make a significant contribution to AS without ID. Less deleterious variants such as inherited missense variants are more difficult to validate (because they require much larger case-control samples) but may contribute to AS in individuals of average intelligence, which is the category of individuals showing the strongest sex bias in AS [67]. For example, Berkel *et al*. identified seven missense variants at highly conserved positions in *SHANK2* only in AS individuals, most of which were transmitted by unaffected mothers [68].

Mutations present in AS males with a low IQ overlap with those found in females but not with those found in AS individuals with a high IQ, demonstrating that sex ratio bias in AS mostly involves high-functioning individuals [67]. Therefore, the AS sex ratio cannot be explained by a genetic mechanism resulting from severe loss of function mutations found in AS individuals with a low IQ, but rather by factors present in AS individuals without ID, in whom severe loss of function mutations have not been found and SIS and PP are consistently reported.

#### Sex differences in synaptic plasticity observed in animal models of AS-associated genes and insults

Although animal models far from capture the complex and heterogeneous human autistic features and are mostly based on syndromic autism with ID, some of them reveal sex differences in synaptic plasticity and thus provide important information about the underlying pathogenic pathways of AS. Furthermore, they constitute promising models to fill the gap between genetically-triggered microstructural alterations and regional plasticity. Indeed, a murine model of autism, known for its structural and connectomic particularities (cortical thickening and loss of the corpus callosum connections), demonstrated shifts in the location of two functional sensory regions [69].

#### Fmr1 knockout mice

Fragile X syndrome (FXS) is the most common form of inherited ID and a leading cause of ‘syndromic’ autism with ID [70]. FXS occurs in both sexes, but women are usually less affected than men because of compensation by the normal *FMR1* gene on the second X chromosome. This disorder is caused by the loss of the X-linked *FMR1* gene product, fragile X mental retardation protein (FMRP), a mRNA-binding protein involved in translational regulation that plays a crucial role in brain development, synaptogenesis, and synaptic pruning [21,71]. FMRP represses the synthesis of proteins required for protein synthesis-dependent synaptic plasticity and acts as a switch to enable translation in response to synaptic signals [72]. *Fmr1* knockout (KO) mice (mostly males) exhibit abnormal synaptic plasticity. long-term depression (LTD) of synaptic transmission is enhanced in the hippocampus [73] and the cerebellum [74], whereas long-term potentiation, (LTP) the most studied form of plastic strengthening of synapses, is impaired in the cortex [75-79] and the amygdala [77]. The abundance of calbindin in the dorsal thalamus is substantially lower in *Fmr1* KO male mice than in female *Fmr1* KO or control mice [80]. Neurons in the dorsal thalamus are glutamatergic, and low levels of calbindin may affect the functional properties of the circuits in which they are involved, for example by inducing long-term changes in the efficacy of excitatory synapses. Thus, *Fmr1*KO mice show male-specific abnormalities of synaptic plastic reactions probably because of the loss of *Fmrp* and the lack of gene dosage compensation from a second X chromosome.

FXS is therefore an example of differential plasticity between the sexes triggered by a genetic insult, where males are more susceptible to develop the syndrome than females. The compensatory role of the intact X chromosome in females does not explain why autism sometimes occurs in combination with FXS. Thus, other sex-related differences in plasticity may act in combination with the loss of FMRP to determine whether FXS occurs alone or with autism.

#### SHANK genes animal models

*SHANK* genes code for large synaptic scaffold proteins and bind to many proteins at the post-synaptic density (PSD) of excitatory synapses. Numerous deleterious mutations have been discovered in *SHANK* genes in AS and other neurodevelopmental disorders. Recent meta-analyses and review articles have shown that there is a gradient of severity in cognitive impairment, as well as sex ratio, in AS individuals carrying mutations in *SHANK* genes. Mutations in *SHANK1* are only present in males with a normal IQ and autism. Mutations in *SHANK2* are found in AS patients with mild ID, with a high male to female ratio. Finally, mutations in *SHANK3* are found in AS individuals with moderate to profound ID, with an almost equal male to female ratio [81]. *Shank1* mice show altered post-synaptic density (PSD) protein composition, small dendritic spines, and weak basal synaptic transmission [82]. Spatial learning and memory are better in *Shank1*−/− male mice than in Shank1−/− female mice [83], indicating a hyperplastic reaction in memory formation in males. Deletion of *Shank2* in mice results in an early, region-specific upregulation of ionotropic glutamate receptors at the synapse and high levels of *Shank3*. Moreover, *Shank2*−/− mutants exhibit fewer than normal dendritic spines, impaired basal synaptic transmission, and disrupted postsynaptic currents [84]. Mice with *Shank3* gene deletions exhibit self-harming behavior, repetitive grooming, and deficits in social interaction. Cellular, electrophysiological, and biochemical analyses have uncovered defects at striatal synapses and cortico-striatal circuits in *Shank3* mutant mice [85]. Therefore, knockout-mice models of *Shank* genes indicate that each gene plays an important and sex-specific role in synaptic plasticity. However, these sex differences may not be observable in KO models because of the major deleterious effects of these mutations.

#### The VPA animal model

The valproic acid (VPA) model [86] is the most extensively studied animal model of autism (for a review see [87,88]). This model is insult-based and does not involve major genetic modifications; therefore, it allows the investigation of synaptic plasticity pathways in a wild-type background. A single prenatal injection of VPA on embryonic day 11.5 significantly stimulates the local recurrent connectivity of neocortical pyramidal neurons but limits the strength of the connections [89]. Hyperconnectivity and hyperplasticity in the medial prefrontal cortex, the somatosensory cortex, and the lateral amygdala have also been observed in this model [90,91]. Synaptic plasticity experiments between pairs of pyramidal neurons have revealed the existence of a strong postsynaptic form of LTP in mice exposed to VPA *in utero* [89]. Rinaldi *et al*. [92] subsequently reported the selective overexpression of two subunits of the NMDA receptor and the commonly linked kinase calcium/calmodulin-dependent protein kinase II, indicating that VPA strongly stimulates NMDA receptor-mediated transmission and promotes plasticity in the neocortex. Mowery *et al.* [93] also found higher cell counts in male than in female VPA rats, indicating a stronger but more aberrant local plastic reaction to VPA in males.

Sex-specific behavioral and immunological alterations have been observed in the VPA model [94]. VPA promotes neural progenitor-cell proliferation and induces macrocephaly in rat brains *via* a mechanism involving the GSK-3β/β-catenin pathway [95]. In particular, male-specific alterations in excitatory post-synaptic development and social interactions have been observed [96]. Moreover, VPA-exposed male offspring show hyperactivity and strongly impaired social interactions whereas the phenotype of female offspring is less pronounced. The expression of the GABAergic neuronal marker GAD is low and that of the glutamatergic neuronal marker vGluT1 is high in both male and female rats, but post-synaptic markers such as PSD-95 and α-CAMKII are strongly expressed only in male offspring. Electron microscopy detected a higher than normal number of post-synaptic compartments in males but not in females at 4 weeks of age, suggesting that the altered glutamatergic neuronal differentiation leads to perturbations of post-synaptic maturation only in male offspring prenatally exposed to VPA. Male VPA-exposed rats are more sensitive than females to electric shock, consistent with the large size of the post-synaptic compartment in males [96].

Male rats exposed prenatally to VPA frequently develop an abnormally large number of glutamatergic synapses. Kim *et al.* [97] recently showed that methyl-CpG-binding protein 2 (MeCP2) influences sex differences in postsynaptic development in the VPA animal model of autism. MeCP2 loss-of-function causes Rett syndrome in girls [98]. VPA exposure leads to male-specific abnormalities in the timing of excitatory glutamatergic synaptic protein expression and results in male-specific attenuation of MeCP2 expression both in the prefrontal cortex of offspring and in neural progenitor cells (NPCs). Furthermore, small interfering RNA (siRNA) against *Mecp2* inhibits Mecp2 expression in male-derived NPCs, resulting in the induction of postsynaptic proteins such as PSD95 but has no effect on female-derived NPCs. Thus, low *Mecp2* expression in males is involved in the abnormal development of glutamatergic synapses, which may explain why males show more abnormalities than females in VPA animal models of AS. Protective effects of estrogen and progesterone and sex-related differences in the development and/or functioning of neurotransmitter systems may also play a crucial role in the protection of female rats from VPA-induced aberrations [94].

### Sex differences and autism-specific aspects of brain architecture and function

#### Typical structural and connectome sex differences

#### Developmental changes in gray matter structure

The cortex attains its maximal complexity just after birth, but soon undergoes global thinning and a decrease of gyrification until early adulthood [99]. This global pruning is accompanied by the building and reinforcement of neural circuits through experience-dependent neurogenesis and synaptogenesis [100]. No sex differences have been reported in the developmental trajectories of overall cortical volume, thickness, and surface area [101]. By contrast, sex differences have been observed in specific regions. A recent longitudinal study examined deep changes of gyrification occurring in infants from birth to 2 years of age [102]. Sex differences were observed at birth around the calcarine fissure and at 2 years of age in the left paracentral cortex. Large increases of gyrification occurred in multimodal association cortices (prefrontal, temporal, inferior parietal, and precuneus) which, in contrast to unimodal regions, were not mature at birth. These regions are more likely to be reshaped by environmental factors because they are less genetically constrained than primary regions [103] and also because immature systems tend to incorporate information from the environment and from personal experience into their structure and function [100,104]. Other studies have shown that cortical modifications occurring from childhood to adulthood differ according to sex [105,106]. Males show a larger decrease of gyrification than females specifically in the right pre-frontal cortex. Thickness changes are even more dependent on sex; in females, temporal and frontal lobes show a high rate of thinning whereas in males, thinning is faster in the occipital lobe [105]. Raznahan *et al*. [105,106] found that most frontal regions matured earlier in females than males, whereas the opposite was true for a large posterior temporal and parieto-occipital area. These late-maturing regions showed accelerated thinning during adolescence corresponding to networks underlying cognitive functions for which the ‘late’ sex tended to perform less well. These two results reveal the sex-related differences of developmental trajectories of the frontal brain as opposed to the perceptual associative areas. In addition, the volume of the mature human brain is smaller in females than in males, but females display greater gyrification and thickness [107-109]. These two latter features may merely reflect the optimization of space in a smaller intra-cranial volume in females; however, they may also be related to functional sex-specific cognitive strengths because gyrification reflects microstructural organization. Taken together, these findings demonstrate the existence of developmental time windows during which particular brain regions exhibit high levels of plasticity in a sex-dependent fashion.

#### Connectome and white matter findings

**Figure 1:**
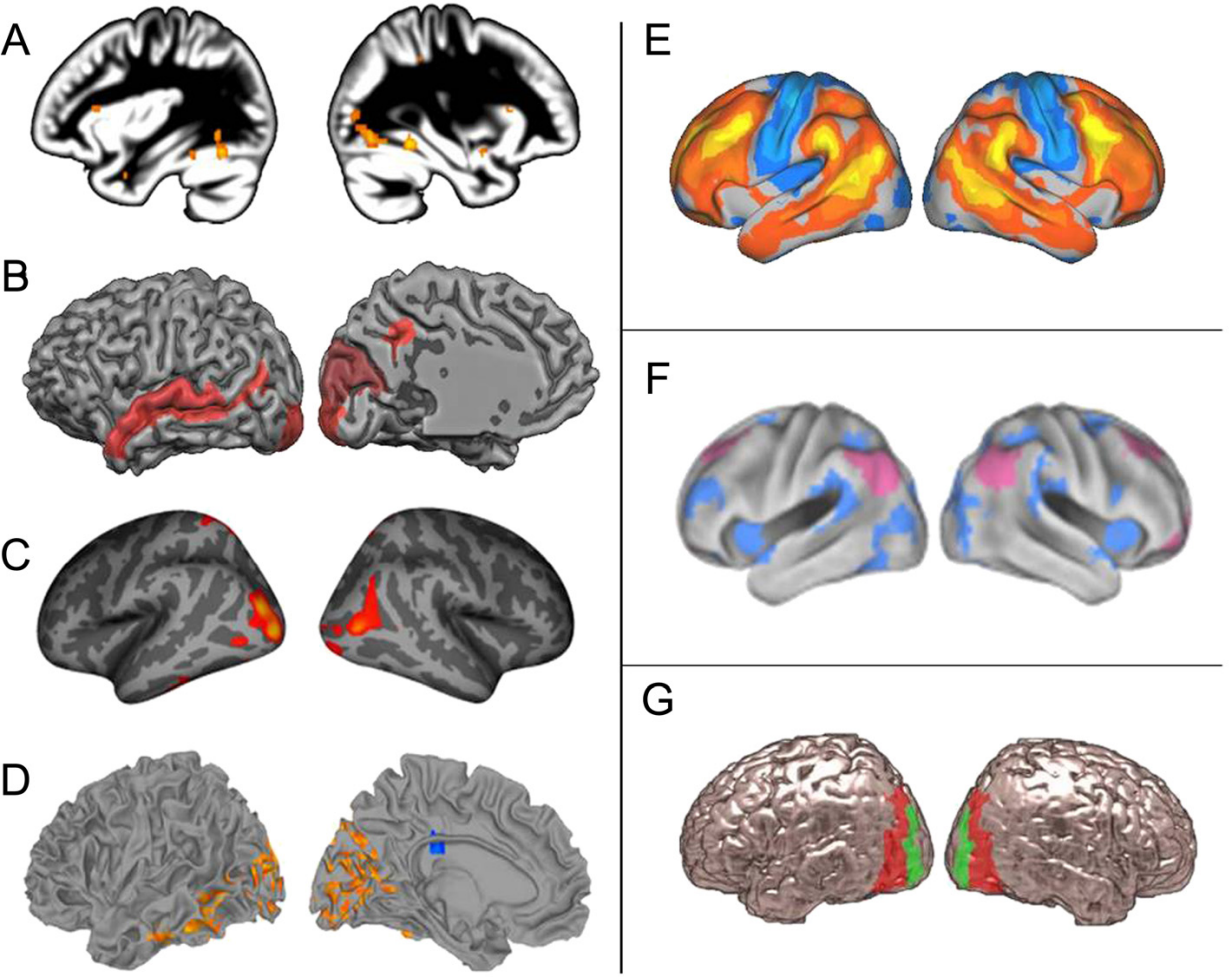
Convergence between functional **(A)**, structural **(B,** **C)**, and connectivity **(D)** regions of interest in autism and regions of maximal variability **(E)** and sexual dimorphism **(F)** in typical individuals in a visual associative area **(G)**. **(A)** Regions showing more activity in autistic individuals than in non-autistic controls when processing visual information. Qualitative meta-analysis, whole brain FDR corrected [16]. **(B)** Regions showing greater cortical gyrification in autistic individuals than in non-autistic individuals. The warmer the color, the greater the significance of the group differences [152]. **(C)** Regions showing higher thickness in autistic versus non-autistic individuals. More than 1,000 brains analyzed, FDR corrected [151]. **(D)** Regions of enhanced resting-state local connectivity density in autistic individuals. Warm colors show the regions with greater connectivity in the autistic individuals than in non-autistic individuals, and cool colors regions of lower connectivity [134]. **(E)** High inter-individual variability in resting-state functional connectivity in non-autistic individuals. Values above or below the global mean are displayed in warm and *cool* colors, respectively [17]. **(F)** Regions of higher resting-state functional connectivity in males (*blue*) and females (*pink*). Seed-based analysis on more than 1,000 brains corrected with Gaussian random-field theory [118]. **(G)** Bilateral visual associative cortex: Brodmann Areas 18 (*green*) and 19 (*red*).

Boys have a larger callosal volume than girls at birth [110] and faster or more pronounced increases in white matter volume during development [111]. Sex differences in white matter fibers were recently studied in a large sample of individuals between 8 and 22 years old [112]. This study shows that cortical wiring differs between sexes; intra-hemispheric connections were dominant in males whereas inter-hemispheric connections dominated in females. Male networks showed a highly modular organization, with strong local cross-talk, whereas female networks were more strongly connected between lobes, although the inverse pattern was found in the cerebellum. The modular organization of the male connectome was detected from early adolescence, starting in the temporo-parietal regions and later spreading from the occipital to posterior frontal regions. Recent functional connectivity results confirm these findings and show that females have greater overall connectivity density than males at rest, meaning that each region is connected to a higher number of nodes in females than in males [113]. Moreover, connectivity inside functional networks (that is, brain regions that may be distant from each other but work together to perform a particular function) is higher in females than in males (males show instead more extensive between-network connectivity) [114]. This indicates that female functional networks tend to be more segregated, which is also the case for structural connectivity [115]. Nevertheless, other results indicate that local connectivity is more efficient in females than in males [116,117]. Regional sex differences in connectivity efficiency largely involve the association cortices [117]. One study investigating task-related connectivity found a differential relationship in girls and boys between connections in the auditory system and IQ. In boys, intelligence was related to the connection between Broca’s area and auditory processing regions, whereas in girls it involved the connection between the right posterior temporal gyrus and other auditory areas [115]. Conversely, a meta-analysis based on three different measures of functional connectivity and using data from over 1,000 non-autistic individuals demonstrated the existence of region-specific sexually dimorphic connectivity; for instance, males have higher connectivity in occipital and temporal regions than females [118] (see Figure 1F).

#### Autism-specific aspects of brain structure, connectivity and development overlapping with sex differences

#### Connectivity findings

Alterations in network connectivity are consistently found in autistic individuals (as recently reviewed in [119]). Compromised white matter integrity (see [120-122]) coupled with long-range hypo-connectivity counterbalanced by local hyper-connectivity [123-125] are commonly found in autistic individuals. However, studies examining connectivity in autism report some inconsistent results and their conclusions may differ according to the method used to assess connectivity [126]. Consequently, we only report here recent results that parallel sex differences reported in 2.1 or related to AS hyper-functioning. Based on previous results showing that thickness correlations between regions are related to anatomical connections, Shi *et al*. [127] found that in AS children, thickness correlations between regions of the same functional network were lower whereas those outside networks were higher than in typically developing children. This finding was replicated by Zhou *et al.* [128] who further reported that functional connectivity between some individual regions is low in AS, but the overall efficiency of networks in AS and non-autistic children according to functional activity is similar. Thus, alternative connections in the autistic brain enable atypical, although effective cognition. Local connectivity alterations are certainly related to autism. For instance, only individuals with autism, with or without tuberous sclerosis, show a low ratio of long over short-range coherence of EEG connectivity, which is absent in individuals with tuberous sclerosis alone [129].

Measurements of resting-state functional and structural connectivity indicated that the brain networks of AS individuals may be less functionally differentiated than those of non-autistic individuals [126,130]. However, studies of task-related functional connectivity do not support this statement. For instance, both short and long-range occipital hyper-connectivity were observed in AS individuals during visual search, a task related to a hyper-functioning cognitive domain in autism [131]. In studies examining regional measures of functional connectivity, hyper-connectivity has been often reported in autistic individuals in regions related to the visual system. In AS individuals, the visual cortex has many internal connections (Figure 1D, [132-134]) and is highly connected to the frontal lobe [135,136]. Furthermore, AS individuals show high connectivity between associative perceptual regions such as the parietal and temporal lobes [137] and inside the medial temporal cortex [138].

Low inter-hemispheric functional connectivity (probably resulting from low callosal volume [139]) has been found in AS individuals [140]. Rudie *et al*. [141] examined functional connectivity and reported that both the overall and local ‘efficiency’ of autistic brain networks was low, confirming that the segregation of brain systems is less defined in AS than in non-autistic individuals. However, their results regarding anatomical connectivity (measured by DTI) revealed a different pattern, which demonstrates the importance of comparing similar measures when analyzing connectivity. In non-autistic individuals, the brain tended to switch from a modular to a more globally efficient structural organization during adolescence, whereas AS networks tended to keep their modular organization. An EEG connectivity study also reported that the organization of the brain of AS individuals was highly modular [124].

#### Developmental findings

General cerebral overgrowth of gray and white matter has been consistently observed in autistic children during the first years of life (for a review see [142]), as recently illustrated by measurements of high surface area in AS [143]. This early growth stage is very relevant; the peak of synaptogenesis occurs at this stage [100] where the first observable structural sex differences emerge. In addition, it is also the stage at which the first detectable clinical and behavioral signs of autism appear [144]. Perceptual signs such as long visual fixations are among the first to manifest at around nine months of age [145], and brain overgrowth peaks at the same time [146]. The negative social and speech symptoms of autism are detected on average around one year later. Courchesne *et al*. [147] further showed that this overgrowth mainly involves frontal and temporal cortices, whereas parietal and occipital cortices were largely unaffected. However, during adolescence this overgrowth was followed by an accelerated decrease of volume. Other studies also have reported perturbations of gray matter development in AS individuals during childhood and adolescence involving either precocious maturation [148] or accelerated decrease in the volume of particular regions, with the most striking differences localized to the occipital cortex [149]. Taken together, the developmental course of structural alterations reported in autism show that the manifestations of autism mainly involving overt socio-communicative ‘negative’ signs are visible after a period of structural overdevelopment.

#### Structural findings in the mature autistic brain

Only one study to date has compared anatomical differences between both males and females and autistic and non-autistic individuals [150]. This study focused on local volumetric measures and found several regions showing volume differences between the sexes. Autistic and non-autistic individuals showed few volumetric differences and there was no overlap between atypical structures in autistic males and sexually dimorphic structures in controls, although gray matter showed small overlap between autistic females and sexually dimorphic structures in controls. This overlap was more pronounced in white matter. Cerebral volume is perhaps not the best measure to assess similarities between sexually dimorphic and autism-specific structural alterations.

Examination of the structure of the mature cortex indicates that the occipito-temporal and parietal regions are thicker (Figure 1B) [151], and gyrification in the precuneus and occipito-temporal areas is more pronounced in autistic than in non-autistic individuals (Figure 1C) [152]. Voxel-based morphometry meta-analyses have also revealed volume abnormalities in regions including the occipital, parietal, and temporal lobes [153] together with low frontal and high occipital gray and white matter volume in autistic individuals [154]. Additionally, the intra-parietal sulcus and the parietal operculum have been found to be deeper in individuals with Asperger syndrome and autism, respectively, than in controls [155]. Differences in geometrical measurements have also been found in the central, frontal, medial, and intra-parietal sulci and in the developmental trajectory of these regions between autistic individuals and controls [156].

#### Topographical convergence between sexually dimorphic, highly variable brain regions and those showing differences between autistic and non-autistic individuals

**Figure 2:**
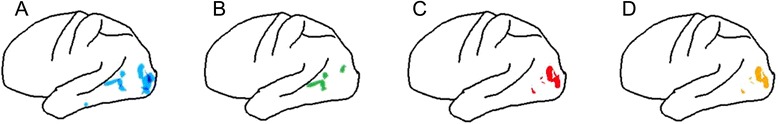
Topographical overlap between functional, structural, and connectomic particularities in the autistic left-hemisphere **(A)** and regions of high variability **(B)** and sexual dimorphism **(C)** in the general population in a visual associative area **(D)**. Patterns of this schematic representation were obtained by manual alignment, distortion and superimposition of the results from the different relevant studies presented in Figure 1. **(A)** Overlap between two (*light blue*) or more (*dark blue*) autistic particularities out of four studies reporting higher thickness [151], gyrification [152], functional activity [16], and connectivity [134] in autism (left panel in Figure 1). **(B)** Overlap between the autism-specific region defined in A and regions of high inter-individual variability in connectivity ([17] and Figure 1E). **(C)** Overlap between the autism-specific region defined in A and regions of higher connectivity in males ([118] and Figure 1F). **(D)** Overlap between the region defined in C and the visual associative regions (Brodmann Areas 18 and 19, Figure 1G).

In the two previous sections, we listed the striking similarities between male-female and AS-non AS differences in brain imaging areas that are related to plasticity mechanisms, and proposed that structure and connectivity patterns in the mature brain reflect previous experience-dependent plasticity. Although indirect, this evidence suggests that the idea of a central role of cerebral plasticity in AS male bias, based on genetic and animal findings, can be extended to human cognition. Indeed, qualitative examination of structural and functional differences between autistic and non-autistic individuals indicates a striking overlap between regions of major anatomical, connectivity-related and even functional (Figure 1A [16]) alterations in autistic individuals, and regions of cross modal plasticity in non-autistic, sensory-impaired individuals [157]. These overlapping regions, all of which involve perceptual associative areas, also overlap with regions showing (1) high variability of functional activation in autistic individuals [13]; (2) high inter-individual variability in resting-state functional connectivity in non-autistic individuals of either sex (Figure 1E [17]); and (3) higher connectivity in males than in females (see Figures 1 and 2). This overlap is indirect, although promising evidence of the following notion that is central to the TTT model [20]; enhanced cognitive performances in autistic individuals result from an enhancement of experience-dependent plasticity mechanisms, targeting perceptual associative regions. This model supports the hypothesis that early overgrowth, supposedly hyperplasticity originating prenatally, prevents the experience-dependent shaping of the brain in frontal regions, resulting in (and subsequently further reinforcing) autistic social impairments [142]. The implicated regions overlap with the sexually dimorphic regions in non-autistic individuals, in particular, the developmental opposition between ‘social’ (frontal and superior temporal) and perceptual (parieto-occipital and inferior temporal) regions. A sex-dependent plasticity imbalance between these regions may thus be central to the onset of autism.

Sexual divergence of developmental trajectories also suggests that the plastic reaction happens in different regions in boys and girls, resulting in a different autistic phenotype in men and women. This explains why males and females may be diagnosed according to different symptoms and/or using different weights attributed to the three diagnostic areas [158,159]. A meta-analysis of studies investigating sex differences in ASD symptoms concluded that males and females had similar communication and social symptoms, but girls showed fewer repetitive, restricted behaviors [160] and interests (RRBI) than boys. This finding is most frequently reported in studies including adolescents [161] and adults of typical intelligence [162], suggesting that compensatory social communication strategies develop over time, especially in girls [163].

### Hormonal effects involved in sexual dimorphism of brain regional plasticity

We will now review sex differences in fetal hormonal effects, memory formation, stress response plasticity, and reaction to brain injury in adults. These sex differences result from both hormonal and non hormonal sex-specific mechanisms of brain plasticity, which lead to a sex difference in the adaptive response of the brain to several types of injury, regardless of age.

#### Fetal hormonal effects and their relevance to the onset of autism

The brain is influenced by maternal, placental, and fetal hormones during early development [164]. Developing testes are more active than ovaries, resulting in higher levels of circulating testosterone (T) and estradiol in male than in female fetuses. Consequently, many studies have focused on the effects of these masculinizing hormones on brain development, despite the involvement of other hormones such as progesterone. Prenatal hormones thus affect neuronal cell proliferation, localization, apoptosis, and synaptic plasticity in a sexually dimorphic manner. Currently, no direct evidence in humans exists to show that these hormones contribute to sex differences in cerebral regions; however, studies in rodents and other animal models have shown that several regions showing sex differences, including the sexually dimorphic nucleus of the preoptic area [165] and hypothalamic structures, are organized prenatally by sex hormones. Some of these prenatal effects are irreversible and some of them only appear at adolescence following their reactivation by increases in steroid hormone concentrations (for a review see [164,165]).

According to the extreme male brain (EMB) theory, the effect of fetal testosterone (fT) may account for male bias in autism ([166,167]; see Table 1). This theory posits that female brains are optimized for ‘empathizing’ whereas male brains are optimized for ‘systemizing’. Empathizing refers to the capacity of identifying, understanding, and reacting correctly to another individual’s thoughts and emotion, and systemizing is the ability to understand and predict the functioning of a law-driven system. Here, the autistic brain is an example of a hyper-systemizing brain which has been masculinized by high levels of fT during gestation [7]. Preliminary evidence of high levels of fT (and other androgenic hormones) in males with autism was only found recently [168]. These overall differences were detected at the group level. Androgen levels in individuals with autism, Asperger, or pervasive developmental disorders largely overlap with those of control individuals, suggesting that high fT is a susceptibility factor and not necessarily a direct cause of autism. Autistic females are also more likely to develop steroid-related conditions, such as polycystic ovary syndrome, than non-autistic females, further suggesting that exposure to abnormal levels of androgens is associated with autism [169]. However, a recent comprehensive review found only a weak link between steroid-related disorders and autistic traits and showed that the prevalence of autism is not high in clinical populations exposed to high androgen levels [164].

The complex role of fT in autism has been further documented in a rat model in which a hyper-androgenic environment was created by high T levels during pregnancy [170]**.** This impaired the vocalization behavior of rat pups of both sexes. In adolescence, the female offspring spent less time engaging in social interactions and exhibited impaired heterosexual interactions as adults. Although the affected behaviors can be classified as social, this model does not mimic the complexity of autistic features in humans. Furthermore, the social impairments in this rat model disappeared in adulthood. Nevertheless, these results indicate the sexually dimorphic consequences of high T levels. Thus, similar hormone levels probably have differential effects in males and females, mainly because of the compensatory reduction of T production in the male fetus in response to high maternal exposure, which cannot occur in the female fetus. Alternatively, in males, high T levels may not be sufficient to produce autistic-like behaviors and other mechanisms may be necessary. In summary, interactions between hormones and genes should be considered in the physiopathology of autism. For instance, androgens and estrogens differentially regulate the *RORA* gene, a candidate susceptibility gene for autism, the expression of which is low in the frontal cortex of autistic individuals [171]. The product of this gene, which promotes the conversion of T into estrogen, also acts through co-activators, demonstrating the complexity of the gene/hormone interactions [172].

#### Sex differences in memory formation and stress response plasticity driven by hormones: the hippocampus

Sensory experience drives the formation and elimination of synapses resulting in experience-dependent plasticity [173]. Plasticity differences between the sexes have been extensively studied in the hippocampus. For instance, Ca2+/calmodulin kinase alpha (CaMKKa) and beta (CaMKKb) are required for memory formation in male, but not female mice. CaMKKb activates the ubiquitous transcription factor regulator cAMP response element-binding protein (CREB) to regulate the formation of spatial memory specifically in males, thus resulting in sex differences in the activation of gene transcription. Conversely, estrogen promotes the formation of dendritic spines on CA1 pyramidal cell dendrites in adult female rats only [174]. Furthermore, estrogen has various actions on adult hippocampal neurogenesis, synaptic plasticity in the hippocampus, and cognition in female rats [175]. Estradiol synthesis is associated with synapse density in *in vitro* cultures of rat hippocampus from either sex, whereas synaptic loss is induced by estrogen depletion *in vivo* specifically in females [176]. These discrepant findings are explained by a sex-specific link between hippocampal plasticity and the concentration of circulating hormones.

Brain-derived neurotrophic factor (BDNF), one of the key molecules modulating brain plasticity, induces long-lasting potentiation of synapses during specific learning and memory processes [177]. BDNF interacts with the major metabolite of T, 17β-estradiol, in mossy fibers during normal hippocampal function [178]. Transcripts of BDNF and two CaMKKb-regulated genes are upregulated in wild-type male, but not female mice, after contextual fear conditioning [179,180]. In mossy fibers, 17β-estradiol upregulates BDNF synthesis in adult female rats, whereas T impairs BDNF expression *via* tonic suppression in adult male rats [181]. Strong excitability associated with high levels of BDNF in mossy fibers in females may facilitate the normal functioning of the CA3 area. The role of interactions between androgens and BDNF in the maintenance of neuronal populations and plasticity has been acknowledged in several sexually dimorphic nuclei in various animal models [182]. Therefore, the sex-specific regulation of BDNF expression strongly suggests that the sexes use distinct forms of synaptic plasticity (for example, mediated by different molecular actors) during contextual memory formation [183].

The effects of stress on hippocampal neurogenesis and synaptogenesis are also sexually dimorphic. Chronic restraint stress produces atrophy of the dendritic tree of CA3 neurons, exclusively in the apical field in males and only in the basal field in females [184]. Chronic stress impairs cell proliferation and survival [185] and strengthens presynaptic inputs in males [186], whereas the inverse pattern occurs in females. Chronic stress in early life has lasting consequences on hippocampal structure and function in mice and suggests that male mice are more susceptible than females to early stress [187]. Therefore, it seems that males and females use distinct molecular mechanisms to learn from the same tasks. Alternatively, sex-dependent strategies in the same learning situation may activate distinct molecular processes [188,189] and result in superior experience-dependent structural modifications in males [190,191].

#### Sex differences of regional brain plasticity after brain damage in adulthood

Females are protected against stroke and its inflammatory effects both at a young age and post-menopause, when sex differences in circulating hormones are minimal [192]. However, women seem to be at a disadvantage in particular conditions where brain repair or reorganization is an important component of post-acute phase recovery, such as stress response plasticity, the pre-clinical phase of Alzheimer’s disease [193], functional recovery after trauma or ischemic stroke [194], and multiple sclerosis [195,196]. In these conditions, the outcome of female patients is worse than that of male patients.

#### Ischemic stroke and NOS1

Nitric oxide synthase-1 (NOS1) is an enzyme involved in several forms of plasticity including hippocampal-dependent learning and memory, experience-dependent plasticity in the barrel cortex, and LTP in the hippocampus and neocortex. LTP is absent in male but not female αNOS1 knockout mice, indicating that residual LTP in females is not nitric oxide-dependent. Experience-dependent potentiation resulting from single-whisker experience (removal of all but one whisker from one side of the face) is significantly impaired in male αNOS1 knockout mice but does not affect females, suggesting that cortical plasticity relies more on NOS in males than in females. Therefore, synaptic neocortical plasticity mechanisms differ between males and females, including both basic plasticity induction pathways and the ability of plasticity mechanisms to compensate for the loss of αNOS1 [197].

#### Mild traumatic brain injury

Sex differences in the functional outcome of traumatic brain injury (TBI) have long been recognized [198]. In animals, females exhibit lower TBI-related mortality, morbidity, and behavioral deficits than males [199,200]. According to the gonadal steroid hypothesis, gonadal hormones, such as progesterone, confer a prophylactic effect, thus limiting the severity of injuries and facilitating recovery in females [201]. However, the role of biological sex in human TBI is less clear, with conflicting reports regarding mortality and morbidity [202,203]. Mild TBI and sport-related concussion in particular provide a better model than severe TBI to understand the influence of sex on TBI outcome. Females exhibit post-concussion syndrome more frequently than men [198] and take longer to return to school/work following injury [204]. Furthermore, sport studies report that TBI cognitive performance is poorer in women than in men [205,206] and show that women more frequently experience concussion-related symptoms [206].

Studies examining concussive injuries of various origins have identified hormone disruption as a key factor underlying sex differences in the outcome of concussion [198,207]. One-month post injury, women taking birth control report fewer neuropsychological and neuropsychiatric symptoms than other women and men [198]. In addition, women injured during the luteal phase of menses report more somatic symptoms and a lower quality of life than those injured during the follicular phase or on birth control [208]. However, not all studies conclude that men have a more favorable outcome than women following concussion [209,210]. Nonetheless, most studies indicate that women exhibit poorer outcomes than men and implicate hormonal disruption as a key factor accounting for sex-related differences in TBI outcomes.

#### Multiple sclerosis

Multiple sclerosis (MS) involves the destruction of the nerve cover in brain and spinal cord white matter, developing either continuously without remission or to complete remission interspersed with successive attacks. Compensatory plasticity plays a major role in clinical relapses in MS. The sex ratio favoring women has been increasing from 2:1 to 3:1 in the past few decades [196].

Compensatory plasticity in MS can involve regional rededication (for example, the hyper-activation of alternative regions during the active phases of MS) or microstructural modification (for example, modifications of synaptic strength in intact regions). MS patients with a lateralized motor deficit display high ipsilateral and contra-lateral activity in cortical motor areas, which are less activated or not activated at all in control individuals. The enhancement of activity in cortical motor regions is correlated with brain damage [211,212] and can be seen from the amplitude of low frequency resting state activity [213]. This pattern is partially reversible during the remission phase. Strong LTP in MS patients demonstrates the plastic adaptation of intact neurons. In patients undergoing remission, platelet-derived growth factor (PDGF) is associated with strong LTP and high regional compensation. By contrast, in MS patients not undergoing remission or in those with progressive MS, strong LTP is not detected in intact regions [214,215]. A protective effect of physiological T may be responsible, at least in part, for the low susceptibility of men to MS [196]. However, animal models enabling the effects of sex hormones and chromosomes to be studied separately indicate that genetic sex plays a major role. For instance, genes on the Y chromosome have a protective effect in EAE, an animal model of MS [216].

The relationship between sex differences in functional compensation in adults and during early developmental cortical reallocation remains to be established. Nonetheless, differences in tissue repair support the idea that women are at a disadvantage regarding their plastic reaction to brain injury, as a result of combined genetic and hormonal effects [165]. This may be consistent with preliminary histological findings reporting a larger number of synapses in the temporal neocortex of epileptic male adults with mesial temporal lobe epilepsy (secondary to hippocampal alterations), than in females with the same condition. Although this evidence is indirect and requires further validation, these observations suggest the existence of sex differences in brain plasticity indicative of a low general level of plasticity in females, which may interfere with early cortical reallocations involved in autism.

## Conclusions

Recent studies investigating sex-differences in autism have attempted to distinguish sex differences in diagnosis and phenotype from the search for ‘general models of etiology and etiological-developmental mechanisms’ [217]. With this in mind, we searched for a possible link between enhanced perceptual functioning and the biased sex ratio in autism. We examined studies investigating sex differences in the brain transcriptome, brain imaging data, brain plasticity following trauma or disease in non-autistic individuals, and the association of these differences with comparable common features of AS.

Genetic, transcriptomic, and animal model studies demonstrate the central role of brain plasticity in autism because many mutations involved in AS affect synaptic structure, function, and plasticity. In addition, indirect but nonetheless strong arguments from animal models, brain imaging, development, and studies of the brain transcriptome and plasticity in adulthood suggest that an enhanced plastic reaction is involved in the sex ratio bias in autism. The protective effect of the female sex against these mutations and the presence of sexual dimorphism in pathways involved in synaptic plasticity support the idea that: 1) a similar genetic event may trigger a different plastic reaction in males and females; and 2) plasticity is more likely to be disrupted in males than in females. Other indirect evidence comes from adult sex differences in reconstructive and compensatory brain plasticity.

A review of studies examining sexual dimorphism from brain imaging data of non-autistic and autistic individuals indicates that regions displaying functional, structural, and connectivity enhancements overlap with those in the perceptual (mostly visual) associative cortex that is more connected in males than in females. This convergence, observed in the mature brain, may originate from sexually dimorphic changes occurring during prenatal and early postnatal brain development because gene expression and hormone secretion are highly dependent on biological sex during these periods.

As a new basis for the understanding of sex ratio bias in autism, we now hypothesize that autistic phenotypes result from plastic reactions involving the most plastic, sexually dimorphic brain regions, in individuals whose threshold for this reaction is lowered by genetic or environmental causes. A severe mutation targeting essential synaptic structures may impair synapse formation and trigger a compensatory, abnormal plastic reaction in individuals with a low threshold. In this situation, highly penetrant mutations would cause a monogenic, syndromic phenotype with low IQ whereby the mutated genes are also expressed in other cells and tissues. When less severe mutations occur, the cognitive function of the individual is mainly preserved, while the mutation triggers a mostly typical, but disproportional, plastic reaction. In this paper, we reviewed the arguments indicating that such an occurrence is more likely in males than in females because males have a lower threshold for plastic reactions. The fact that autism-related differences continue to develop throughout life suggests that sexually dimorphic regional plasticity, evident in non-autistic individuals, plays a role in the developmental transformation of the autistic brain. This chain of events applies to autistic individuals with a high IQ, who are disproportionately male, and thus accounts for male-bias in the autistic sex ratio. It also predicts that sex differences in the autistic phenotype are constrained by topographical, chronological, and systemic differences in plasticity.

### Future directions

So far, the explanation for the bias in the autistic sex ratio has been limited to hormone-dependent effects and the questionable similarities between the strong performance of males in some cognitive tasks and autistic talents and PP. The investigation of sex differences involving genetic, *non-hormonal* sex-dependent mechanisms, microstructural experience-dependent plasticity, and importantly, reallocation rules, is clearly a new research avenue, which will extend our understanding of sex bias in autism to factors beyond the hormonal effects documented by the EBM model (Table 1). One of the main assumptions of the hypothesis is that plastic reallocation, even outside the period of hormonal influence, differs according to sex. Thus, it will be important to search for sex differences in functional reallocation following early sensory impairment in non-autistic individuals, as of yet uncharted territory. Another avenue, which may document the “threshold” component of the TTT model is the search for sex ratio or phenotypic sex differences in the most frequent mutations associated with syndromic autism, especially those linked to strong plasticity as documented in animal models.

Synaptic plasticity is the most basic mechanism and best studied form of brain plasticity. Regional plasticity probably involves several molecular plasticity mechanisms at different levels (synaptic, neuronal, regional, and circuitry) and in various forms (functional and structural plasticity). Brain imaging studies have indicated that the changes in AS individuals, in particular those without ID, are regional and compatible with plastic reactions; however, it remains to be demonstrated whether these two levels of enhanced synaptic and regional plasticity are associated in the same person and whether they differ in males and females. Finally, studies assessing domain-specific strengths in women may determine the targets of sex-specific experience-dependent plasticity.

## Abbreviations

AS: autism spectrum
BDNF: brain-derived neurotrophic factor
CaMKK: Ca2+/calmodulin kinase
CNV: copy number variants
CREB: cAMP response element-binding protein
DN: *de novo*
DTI: diffusion tensor imaging
EEG: electro-encephalography
EMB: extreme male brain
FMRP: fragile X mental retardation protein
fT: fetal testosterone
FXS: fragile X syndrome
ID: intellectual disability
IQ: intellectual quotient
KO: knock-out
LGD: likely gene disruptive
LTD: long-term depression
LTP: long-term potentiation
mRNA: messenger RNA
MS: multiple sclerosis
NOS1: nitric oxide synthase-1
NPC: neural progenitor cells
PDGF: platelet-derived growth factor
PP: perceptual peak
PSD: post-synaptic density
RNA: ribonucleic acid
siRNA: small-interfering RNA
RRBI: repetitive, restrictive behaviors and interests
SIS: special isolated skill
SNV: single nucleotide variants
SP: synaptic plasticity
T: testosterone
TBI: traumatic brain injury
TD: typically developing
TTT: trigger-threshold-target
VPA: valproic acid
WES: whole exome sequencing
